# Is bed turnover rate a good metric for hospital scale efficiency? A measure of resource utilization rate for hospitals in Southeast Nigeria

**DOI:** 10.1186/s12962-020-00216-w

**Published:** 2020-07-01

**Authors:** Henry E. Aloh, Obinna E. Onwujekwe, Obianuju G. Aloh, Chijioke J. Nweke

**Affiliations:** 1 Health Economics and Policy Research Unit, Department of Health Services, Alex Ekwueme Federal University Ndufu-Alike Ikwo, Ikwo, Ebonyi Nigeria; 2 Department of Health Administration & Management, Faculty of Health Sciences, College of Medicine, University of Nigeria Enugu Campus, Nsukka, Nigeria; 3 Health Policy Research Group, Department of Pharmacology and Therapeutics, College of Medicine, University of Nigeria Enugu Campus, Nsukka, Nigeria; 4 Primary Health Development Agency, Ministry of Health, Abakaliki, Ebonyi Nigeria; 5 Department of Mathematics/Computer Sciences/Statistics & Informatics, Alex Ekwueme Federal University Ndufu-Alike Ikwo, Ikwo, Nigeria

**Keywords:** Ratio indicators, Resource utilization, Pabon Lasso model, Efficiency, Teaching Hospitals, Southeast Nigeria

## Abstract

**Background:**

Nigeria health sector, like that of other sub-Saharan African countries, increasingly faces critical resource constraints. Thus, there is need to seek for ways of improving efficient use of scarce health resources. The aim of this study was to determine resource utilization rate of teaching hospitals in Southeast Nigeria as a means of estimating their efficiency.

**Methods:**

The study is a longitudinal cross sectional study. It applied ratio indicators and Pabon Lasso model using data on the number of hospital bed, number of inpatients and total inpatient-days from purposefully selected teaching hospitals in Southeast Nigeria to measure efficiency over a period of 6 years (2011–2011).

**Results:**

The hospitals’ mean bed occupancy rate was as low as 42.14%, far below standard benchmark of 80–85%. The mean average length of stay was as high as 8.15 days and observed mean bed turnover was 21.27 patients/bed/year. These findings portrayed high level of inefficiency in Nigeria teaching hospitals, which was further illustrated by Pabon Lasso graph, with only 10–20% of the hospital-years located within or near the efficient zone or quadrant.

**Conclusion:**

The study was able to show that health ratio indicators such as hospital bed turnover rate (BTR) and bed occupancy rate (BOR), as well as patients’ average length of stay (ALS) can be used as tools for assessing hospital performance or its efficiency in resource utilization. Thus, in low and middle income countries where medical record keeping may be inadequate or poor, ratio indicators used alone or with Pabon Lasso graph/chart could be an optional metrics for hospital efficiency.

## Background

Healthcare is one of the most important services provided by the government in every country of the world. It is regarded as a critical resource in the process of economic development. Hence, in both the developed and developing nations, a significant proportion of the nation’s wealth is devoted to the health sector. The health system in Sub-Saharan African countries including Nigeria increasingly face critical resource constraints and this is accounted for by a host of factors such as poor macroeconomic performance, cutbacks in public spending, rapid population growth, various disease outbreaks (e.g. HIV/AIDS, Lassa fever and Ebola fever), and upsurge in diseases such as malaria [1]. The health care system components such as hospitals, in developing countries has for a long time remained under resource constraint and probably inefficient [2].

Performance or efficiency evaluation of hospitals may therefore play a strategic role in healthcare organizations and help address the best use of resources and rationing of demand [3]. Performance evaluation has become central to the concept of quality improvement. It provides a means of defining what hospitals are actually doing and compare it with expected targets [4]. It enhances greater accountability and stimulates continuous quality improvement. This is why WHO Europe Regional Office lunched in 2003 a flexible and comprehensive framework called Performance Assessment Tool for quality improvement in Hospitals (PATH) [5]. Improvement in the efficiency of hospital care is a fundamental aspect of health system strengthening [6]. However, the challenges facing low-income countries is that many keep on struggling without much success to develop and implement feasible strategies to monitor hospital nationally [6].

In sub-Sahara African, hospitals play a key role in the delivery of healthcare services [7]. They also account for the bulk of government’s health sector expenditure, ranging between 45 and 80% in developing countries [8]. Empirical evidence emerging from studies in South Africa [9], in Kenya [10], in Ghana [11] and Namibia [12] indicate a wide prevalence of inefficiency in provision of hospital based healthcare. [13]. In Nigeria, hospitals are perceived to exhibit gross inefficiency [14]. This cut across all the level of healthcare. The tertiary or teaching hospitals are the highest level of healthcare in Nigeria and they take up about 60% of the country’s annual budget on health.

In health systems, the nature of outputs differs from that of other organizations, thus measurements of efficiency are more difficult [15]. In hospital literatures, performance or efficiency are measured using inputs and outputs. Capital input is taken to represent a wide range of manufactured products such as complex medical equipment, buildings, beds and vehicles employed in health care. By nature capital inputs are durable and provide services over a fairly long period of time. Number of beds is the most commonly used variable in hospital efficiency studies. The use of this variable as a proxy for capital inputs has been accepted by researchers [16].

Inpatient services require that patients utilize hospital bed for an overnight stay or for extended treatment over a period of one or more days. A systemic literature reviews carried out by Iranian researchers claimed that out of about 218 indicators used in hospital performance assessment, the most frequently used are average length of stay (ALS) and bed occupancy rate (BOR) [17]. The present study used these two health ratio indicators (ALS and BOR), bed turnover rate (BTR) and turnover interval (TI), as well as Pabon Lasso model to investigate efficiency in resource utilization by Teaching Hospitals in Southeast Nigeria. It is hope that this will proof to be a simple good method for measuring performance of hospitals that treat inpatients. Thus, the study could help develop a tool or metrics for comparing hospital performance [18].

## Methods

The design was cross-sectional and retrospective study. Purposeful sampling method was used to select 3 teaching hospitals that had the requisite health records from Southeast Nigeria. The region is located between longitudes 6° 25′ E and 8° 30′ E, and between Latitude 5° 10′ N and 6° 45′ N. The population of the region is estimated to be 20,683,115 as at 2015 with a growth rate of 2.47% [19]. The hospitals in this study were all University Medical College health institutions, hence they function as teaching hospitals and referral centers, treating mainly chronic and complicated illnesses.

The study used health ratio indicators such as bed occupancy rate (BOR), bed turnover rate (BTR), average length of stay (ALS) and turnover interval (TI) [20] to evaluate efficiency in resource utilization of 3 teaching hospitals that were selected by simple random sampling. Data were collected for a period covering 7 years (2010 to 2016) on the following variables:

- Number of active beds—this refers to number of functional beds for each hospital-year.
- Active beds-days—this refers to the number of functional beds in the hospital for a given period, usually 1 year, and it is obtained as number of active beds multiply by 365 days.
- Number of admissions or discharges in a given year.
- Occupied-bed-days or total inpatient-days, which refers to the sum of total number of days all admitted patients spent in the hospital for a given year.

Using the above variables four ratios or indices were computed as follow [20]:

- $$Bed\,Occupancy\,\,Rate\,\,(BOR)\,\, = \frac{Occupied\,\,Bed - Days\,\,(Total\,\,Inpatient\,\,Days)}{Active\,\,Bed - Days\,\,} \times 100\%$$
- $$Average\,\,Length\,\,of\,\,Stay\,(ALS)\, = \frac{Occupied\,\,Bed\, - Days\,\,(Total\,\,Inpatient\,\,Days)}{Number\,of\,\,Disch\arg es}$$
- $$Bed\,Turnover\,\,Rate\,\,(BTR)\,\, = \frac{Number\,of\,\,Disch\arg es\,(\,or\,admissions)\,\,in\,1\,year}{Active\,\,Beds}$$
- $$Turnover\,\,Interval\,(TI)\,\, = \frac{365}{BTR} - ALS$$

The above formulae were designed into a stata-11 version of Microsoft Excel spread sheet for easier computation.

The study went on to apply Pabon Lasso model or graph to further demonstrate efficiency of the hospital-years, since using any one of the above indicators alone may not sufficiently estimate performance or efficiency of the hospitals. Pabon Lasso Model was originally developed by Pabon Lasso in 1986, and it is a technique used for interpreting and comparing hospital efficiency using three indices [21]. Mathematically, correlations of the 3 indicators were shown by plotting BTR on the y-axis and BOR on the x-axis [22]. And using the average of the two indices (BTR and BOR) 2 perpendicular lines were drawn to divide the graph into four quadrants or zones. The Pabon Lasso diagram obtained was then used as performance assessment tool [23, 24]. Thus, using the four quadrants/zones of the graph, efficiency of various hospital-years were known and the manner with which they utilized available resources are also made clear.

## Results

The record of active hospital beds, as well as number of inpatients and inpatient-days for respective Teaching Hospitals, between year 2010 and 2016 (7 years period), are shown in Table 1. The average (mean) active hospital bed spaces for the hospital-years was 380 beds. The average yearly number of admissions was 8394 inpatients per hospital per year. FETHA had the highest number of admitted patients of 14,037 during the period. While the mean inpatient-days for all the hospitals was 56,912 per year, the highest was UNTH inpatient-days of 85,056 in 2012 (as shown in Table 1 below).

**Table 1:** Descriptive Statistics of Hospital Resources (beds) and Patients admission (2010 – 2016)

Hospital-years	No. of active hospital beds	Active-bed-days	No. of inpatients	Inpatient-days
FETHA 2011	223	81,395	7657	32,751
FETHA 2012	486	177,390	13,400	43,863
FETHA 2013	489	178,485	14,253	53,660
FETHA 2014	512	186,880	16,982	61,388
FETHA 2015	533	194,545	14,885	62,381
FETHA 2016	513	187,245	17,043	59,634
FETHA Mean	459.33	167,656.67	14,037	52,279.5
ESUTH 2010	244	89,060	3359	30,238
ESUTH 2011	244	89,060	1670	20,144
ESUTH 2012	260	94,900	5152	56,672
ESUTH 2013	260	94,900	5433	48,897
ESUTH 2014	260	94,900	4220	54,860
ESUTH 2015	342	124,830	6698	53,584
ESUTH 2016	320	116,800	8094	72,846
ESUTH Mean	275.71	100,635.71	4947	48,177.29
UNTH 2010	424	154,760	6635	66,795
UNTH 2011	424	154,760	8061	84,675
UNTH 2012	424	154,760	8533	85,056
UNTH 2013	411	150,015	8300	85,003
UNTH 2014	411	150,015	5528	55,057
UNTH 2015	411	150,015	4578	43,119
UNTH 2016	411	150,015	7399	67,625
UNTH Mean	416.57	152,048.57	7005	69,618.57
Mean	380.1	138,736.5	8394	56,912.4
Minimum	223	81,395	1670	20,144
Maximum	533	194,545	17,043	85,056

FETHA is Federal Teaching Hospital Abakaliki, ESUTH is Enugu State Teaching

Hospital and UNTH is University of Nigeria Teaching Hospital

**Fig. 1:**
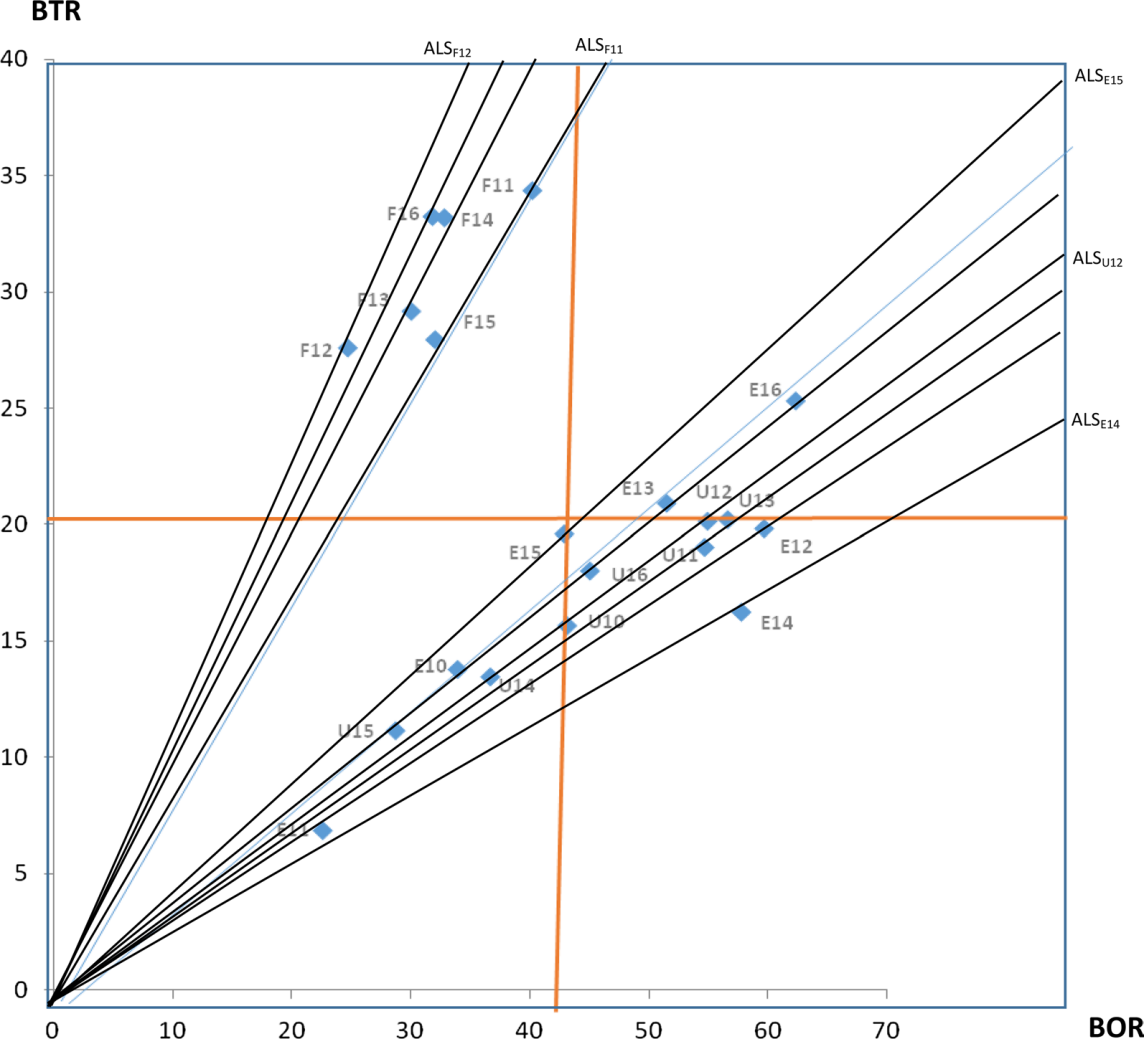
Pabon Lasso graph (BTR: bed turnover rate; BOR: bed occupancy rate; E-i or F-i or U-i represent hospital-year)

Table 2 show that the overall mean bed occupancy rate (BOR) of the hospitals during the period was 42.14%, and the values range between 22.62% and 62.37% among the hospital-years. Mean bed turnover rate (BTR) was 22 patients per bed per year, and the BTR was as low as about 7 patients per bed per year for ESUTH in 2011. The highest value of about 34 patients/bed/year for FETHA was recorded in 2011. Mean average length of stay (ALS) for admitted patients in this study was as high as 8.15 days and the least mean ALS was 3.27 days for FETHA in 2012. The mean turnover interval (TI) for the hospitals was as high as 10.19 days. The shortest average TI for the hospitals was recorded by FETHA as 8.04 days. Thus, apart from long stay of admitted (ALS) patients, all the 3 hospitals exhibited protracted turnover interval (ESUTH—10.72 days and UNTH—11.83 days). Pabon Lasso graph in Fig. 1 emanated from plotting of BTR against BOR.

**Table 2:** Health ratio indicator expressing resource utilization rate of hospitals

Hospital-year	Bed occupancy rate (BOR) in %	Average length of stay (ALS) in days	Bed turnover rate (BTR) in patients per bed per year	Turnover interval (TI in days)
FETHA 2011	40.24	4.28	34	6.35
FETHA 2012	24.73	3.27	28	9.96
FETHA 2013	30.06	3.76	29	8.76
FETHA 2014	32.85	3.61	33	7.39
FETHA 2015	32.07	4.19	28	8.88
FETHA 2016	31.85	3.50	33	7.49
FETHA Mean	31.97	3.77	31	8.04
ESUTH 2010	33.95	9.00	14	17.51
ESUTH 2011	22.62	12.06	7	41.27
ESUTH 2012	59.71	11.00	20	7.42
ESUTH 2013	51.52	9.00	21	8.47
ESUTH 2014	57.81	13.00	16	9.49
ESUTH 2015	42.93	8.00	20	10.64
ESUTH 2016	62.37	9.00	25	5.43
ESUTH Mean	47.27	10.15	18	10.72
UNTH 2010	43.16	10.07	16	13.26
UNTH 2011	54.71	10.50	19	8.69
UNTH 2012	54.96	9.97	20	8.17
UNTH 2013	56.66	10.24	20	7.83
UNTH 2014	36.70	9.96	13	17.18
UNTH 2015	28.74	9.42	11	23.35
UNTH 2016	45.08	9.14	18	11.14
UNTH Mean	45.72	9.90	17	11.83
Mean	42.14	8.15	21	10.19
Minimum	22.62	3.27	7	5.43
Maximum	62.37	13.00	34	41.27

FETHA is Federal Teaching Hospital Abakaliki, ESUTH is Enugu State Teaching

Hospital and UNTH is University of Nigeria Teaching Hospital

The location of each hospital-years in any of the Pabon Lasso quadrants in Fig. 1 describe the level and type of efficiency of such hospital-year. Hospital-years in zone-1 (Low BTR and low BOR) exhibit relatively excess bed supply, less need for hospitalization and low demand/utilization. Zone-ii (high BTO and low BOR) refers to excess bed capacity, unnecessary hospitalization, too many patients being admitted for observation or predominant normal obstetric delivery. The efficient zone - iii (high BTO and high BOR) infers that the hospital-years in this zone had good quantitative performance and small proportion of unused beds. The fourth quadrant (low BTO and high BOR) host hospital-years that had low demand for hospital beds, yet had small proportion of its beds unused. About 20% of the hospital-years were situated in the least efficient quadrant-I, 30% of the hospital-years were located in quadrant-II; while only 10% of the hospital-years were situated in the efficient quadrant-III and 40% of the hospital-years were found in quadrant-IV. It was observed that most of the FETHA hospital-years were located in the second Pabon Lasso quadrants that is characterized by high BTR, low BOR. Two (2) of the hospital-years of ESUTH were located in the efficient quadrant.

## Discussion

It is widely accepted that improved efficiency is one of the four overarching goals of health systems [25]. The World Health Report 2014 estimated that about 20–40% of all health sector resources are wasted [26]. One of the vital approach to reducing resource waste is to enhance efficiency in utilization of available resource [27]. And the starting point in doing so is to undertake performance or efficiency assessment [28]. It is useful in guiding hospital manager at micro level and health policy makers in government at macro level [29].

The present study investigated the efficiency of resource utilization among University Teaching hospitals in Southeast Nigeria; it compared the performance of the hospitals over a period of 7 years (2010–2016). Four ratio indicators: bed occupancy rate (BOR), average length of stay (ALS), bed turnover rate (BTR) and turnover interval (TI) were used to do so. Demonstration of hospital efficiency were elucidated using Pabon Lasso model or graph.

Bed occupancy rate (BOR) is a measure of utilization of the available bed capacity in the hospital, and it indicates the percentage of beds occupied by patients in a given period of time, usually 1 year. It reflects efficiency in the use of hospital beds. And hospital can be said to be operating efficiently at BOR of 80–90% [30]. Among all the hospital-years in the present study the maximum BOR of 62.37% was observed for ESUTH in 2016. Within the seven years period the mean BOR for the hospitals was abysmally as low as 42.14%. Similar study in Uganda hospital over a period of 10 years show that the average BOR was as much as 78.8% [31]. Younsi (2014) in a comparative assessment got a value of 58.1% as mean BOR for 40 public hospitals in Tunisia [32]. A more recent evaluation in Uganda, Sub-Saharan African country, showed average BOR of 49.35% (and BTR of 74.0 times per year and ALS of 3.63 days) [33]. In Iran and other Middle East countries the use of ratio measurement as a means of assessing hospital efficiency is common. Recent studies in different part of Iran showed an average BOR of 65.40% [34], 62.63–69.56% [35], 65.91% [36], as against Iranian national BOR average of 57.8%. The mean BOR obtained from the present study was still below the BOR of 56–61% among public hospitals in Malaysia between 2006 and 2010 [37]. In recent years BOR in countries such as Indonesia range between 55 and 60% in both public and private hospitals, as compared to 80% average for South-East Asian region hospitals [38]. The conventionally suggested benchmark for hospital BOR is 85% [31], signifying that the mean BOR of 42.14% in the present study was relatively very low. And thus, the teaching hospital could be said to have exhibited high level of inefficiency in the utilization of hospital beds during the period under review.

Average length of stay (ALS) refers to the number of days each admitted patient stayed in the hospital. It is often better to compare homogenous group of hospitals that have a similar case-mix. The hospitals studied here were all university teaching hospitals and are known for treating mainly referred and often chronically sick clients. Hospital(s) or hospital-years with shorter ALS than its peers could be regarded to be performing relatively better than those with higher ALS. In this study the lowest ALS was observed for FETHA with a mean ALS of 3.77 days. The explanation for this is either that FETHA was more efficient than the other teaching hospitals in terms of effectiveness in treatment of its patients and in terms of shortening of patients hospital stay. It may also be that FETHA tend to treat more number of acutely ill patients than these other hospitals. The mean ALS for all the hospitals was about 8.15 days. Previous studies on hospitals affiliated to medical school in Iran exhibited mean ALS of 4.1 days [35], 3.21 days [39] and 4.08–4.59 days [40]. This, again show that the hospitals in the present study were less efficient.

Bed turnover rate (BTR) measures productivity of hospital beds, and it represent the number of patients treated per bed in a defined period, usually 1 year. BTR of chronic care hospitals such as orthopedic or teaching hospitals are expected to be lower than those of acute care hospitals. The BTR of the teaching hospitals in this study for the various years were between 6.84 and 34.34 patients per bed per year, with a mean BTR of 21.27 patients per bed per year. This again demonstrate low productivity and high level of inefficiency. The highest value of BTR obtained being that of FETHA with a mean BTR of 30.89 patients/bed per year. The BTR of the hospital-years were all quite low, compared to that of their Iranian counterpart hospitals that are affiliated to medical school, where the BTR were observed to be between 61.10 and 95.54 patients per bed per year [24, 35, 36, 40, 41].

On the other hand, turnover interval (TI) is referred to as measure of the average times or days that hospital beds are unoccupied between successive inpatients. The higher the value of TI of hospital, the less efficient the hospital is. The ideal turnover interval is suggested to be 1–3 days. In the present study all the TI obtained were between 5.43 and 41.27 days with overall mean TI of 10.19 days. This portrays high level of inefficiency or poor performance in terms of hospital bed utilization.

Assessment of hospital performance based on any single ratio indicators may sometime be misleading. Pabon Lasso (1986) had devised a model or graph that makes use of 3 of these ratio indicators (BOR, ALS and BTR) to assess relative performance of hospitals [21]. The plotting of BTR on the vertical axis, BOR on the horizontal axis and the use of mean values of these two ratios to divide the graph into four quadrants is what is known as Pabon Lasso graph [21]. The various hospital-years were located into these four different quadrants. About 20% of the hospital-years were located in the most inefficient quadrants that is characterized by low bed turnover and low bed occupancy rate. The hospital-years located in this quadrant experienced high un-utilize hospital beds. As much as 30% of the hospital-years, mainly from FETHA, were located in quadrant-II. These hospital-years were characterized by high turnover rate, low bed occupancy rate and relatively short stay in hospital by patients. The possible explanation is that there might have been too many un-necessary hospital admission or treatment of many acutely ill patients.

Only 10% of hospital-years were located within the efficient quadrant-III and another 10% were located very close to it. These small fraction of hospital-years exhibited appropriate efficient level or performance. The hospitals enjoyed high bed occupancy and high bed turnover rate in 2013 and 2016. High BTR and BOR implies efficiency or ability of the hospital to efficiently utilize available resources [21, 26]. The study further revealed that the majority (40%) of the hospital-years that were located in quadrant-IV, which were characterized by high occupancy rate, low turnover and long hospital stay. This is not surprising; since the hospitals studied were teaching hospital that usually or often admitted chronically sick patients. Hospital-years in this zone were mainly those of UNTH and that of ESUTH. The findings indicate clearly that half of the hospital-years relatively exhibited high bed occupancy rate (as shown by quadrants-III and -IV). However, in terms of a wholesome efficient performance, the Pabon Lasso graph was able to demonstrate that only as few as 10% of hospital-years were efficient. Thus, the hospitals were unable to efficiently utilize their available capital resources during the period under review (2006–2011). This is in agreement with the result of a recent systemic review of hospital efficiency in East Mediterranean region which show that excess bed supply and inappropriate hospital size are some of the major causes of inefficiency [42].

## Conclusion

Efficiency is widely used in health economics and it simply refers to wise utilization of resources in production of health services. The present study undertook appraisal of teaching hospital performance in the Southeast Nigeria by analyzing their resource utilization rate, covering a period of 2010 to 2016; the findings include: low bed occupancy rate and high average length of stay for the patients. This, is strong evidence of inefficiency among the teaching hospitals in Southeast Nigeria. These observations were emphatically expressed by location of a large proportion (80–90%) of the hospital-years outside the efficient quadrant of Pabon Lasso model.

## Limitations of the study

This study suffers from some limitations upon which future studies should improve upon.

1. Poor quality of health information and record/data keeping among the hospitals is of great concern. In some instance to ensure completeness, data were collected in peace-meal from many departments, instead of the designated Health Record Department.
2. The ratios indicators as a method of measuring hospital performance can only be applied on hospitals that provide inpatient services.

## Recommendations

There is need for various government to make efficiency a policy objective and institutionalize health facility efficiency monitoring and evaluation as a basis for the design and implementation of appropriate policy interventions, and as a means of curbing wastage of health system inputs (WHO, 2014). Health efficiency monitoring should be used as a tool within health management information system (HMIS) of various ministries of health, both at State or Regional level or National level.

## Data Availability

Datasets used and analyzed for the study are available and maybe released by the corresponding author on reasonable request.
